# Supplementary material for: Cross-species activation of hydrogen cyanide production by a promiscuous quorum-sensing receptor promotes Chromobacterium subtsugae competition in a dual-species model
Source: Microbiology (Reading). 2023 Feb 15;169(2):001294. doi: 10.1099/mic.0.001294 (PMC10197875; doi:10.1099/mic.0.001294)
Supplement: Supplementary material 1 [file mic-169-1294-s001.pdf]

C. subtsugae genes activated by C6-HSL

Locus tag corresponding with accession NZ_JAHDTB000000000.1	Predicted gene function	Log_2 fold change compared with no AHLs
KIF53_RS20405	amino acid adenylation domain-containing protein	7.43
KIF53_RS13810	activator protein	7.28
KIF53_RS06805	NAD(P)/FAD-dependent oxidoreductase	6.86
KIF53_RS20410	SDR family NAD(P)-dependent oxidoreductase	6.79
KIF53_RS01865	acyl-protein synthase	6.76
KIF53_RS20400	polyketide synthase	6.51
KIF53_RS20415	acyltransferase domain-containing protein	6.44
KIF53_RS06810	cyanide-forming glycine dehydrogenase subunit HcnA	6.33
KIF53_RS21020	PfaD family polyunsaturated fatty acid/polyketide biosynthesis protein	6.18
KIF53_RS14555	DUF1842 domain-containing protein	6.18
KIF53_RS01860	long-chain-fatty-acyl-CoA reductase	5.94
KIF53_RS21015	DUF3149 domain-containing protein	5.88
KIF53_RS19385	amino acid adenylation domain-containing protein	5.87
KIF53_RS15570	hypothetical protein	5.74
KIF53_RS01870	MdfA family multidrug efflux MFS transporter	5.53
KIF53_RS14680	U32 family peptidase	5.47
KIF53_RS06800	cyanide-forming glycine dehydrogenase subunit HcnC	5.44
KIF53_RS05635	FAD-dependent oxidoreductase	5.42
KIF53_RS16820	M4 family metallopeptidase	5.35
KIF53_RS16340	ornithine carbamoyltransferase	5.23
KIF53_RS19375	ACP S-malonyltransferase	5.23
KIF53_RS05645	FAD-dependent monooxygenase	5.21
KIF53_RS11595	bifunctional phosphoribosylaminoimidazolecarboxamide formyltransferase/IMP cyclohydrolase	5.08
KIF53_RS05655	violacein biosynthesis enzyme VioE	5.04
KIF53_RS13295	hypothetical protein	4.99
KIF53_RS15685	protein	4.97
KIF53_RS14675	SCP2 sterol-binding domain-containing protein	4.94
KIF53_RS19390	acyl-CoA dehydrogenase family protein	4.87
KIF53_RS21040	hypothetical protein	4.86
KIF53_RS16335	arginine deiminase	4.86
KIF53_RS21035	polysaccharide pyruvyl transferase family protein	4.82
KIF53_RS01145	hypothetical protein	4.81
KIF53_RS21030	4'-phosphopantetheinyl transferase superfamily protein	4.74
KIF53_RS21025	alpha/beta fold hydrolase	4.74
KIF53_RS19370	AMP-binding protein	4.73
KIF53_RS06795	ABC transporter substrate-binding protein	4.71

KIF53_RS05640	iminophenyl-pyruvate dimer synthase VioB	4.71
KIF53_RS19365	type I polyketide synthase	4.53
KIF53_RS05650	tryptophan hydroxylase	4.52
KIF53_RS01125	hypothetical protein	4.52
KIF53_RS01855	AMP-dependent synthetase	4.50
KIF53_RS02825	hydrogenase maturation protein	4.48
KIF53_RS15785	methyl-accepting chemotaxis protein	4.45
KIF53_RS14685	U32 family peptidase	4.42
KIF53_RS17975	aspartate carbamoyltransferase	4.34
KIF53_RS16345	carbamate kinase	4.33
KIF53_RS16060	adenylosuccinate lyase	4.32
KIF53_RS06780	ABC transporter ATP-binding protein	4.31
KIF53_RS16225	carbamoyl-phosphate synthase large subunit	4.29
KIF53_RS13290	hypothetical protein	4.26
KIF53_RS06785	ABC transporter permease subunit	4.25
KIF53_RS19360	serine hydrolase	4.23
KIF53_RS08980	acetyl-CoA carboxylase biotin carboxyl carrier protein	4.16
KIF53_RS10155	amidophosphoribosyltransferase	4.12
KIF53_RS19380	AMP-binding protein	4.08
KIF53_RS19395	acyl carrier protein	4.07
KIF53_RS01120	glycosyltransferase family 2 protein	4.02
KIF53_RS03970	type I-E CRISPR-associated protein Cas6/Cse3/CasE	4.02
KIF53_RS13300	peptidase	4.00
KIF53_RS05185	sulfur subunit	3.99
KIF53_RS16220	leucine efflux protein LeuE	3.91
KIF53_RS13815	helix-turn-helix domain-containing protein	3.90
KIF53_RS21000	hypothetical protein	3.82
KIF53_RS11705	helix-turn-helix domain-containing protein	3.74
KIF53_RS05740	hypothetical protein	3.72
KIF53_RS05695	cold-shock protein	3.71
KIF53_RS17075	porin	3.70
KIF53_RS15420	HNH endonuclease	3.64
KIF53_RS15405	hypothetical protein	3.58
KIF53_RS03965	type I-E CRISPR-associated protein Cas5/CasD	3.56
KIF53_RS01130	transketolase	3.54
KIF53_RS00815	type I glyceraldehyde-3-phosphate dehydrogenase	3.52
KIF53_RS18720	dehydrogenase/methenyltetrahydrofolate cyclohydrolase FolD	3.52
KIF53_RS19350	AMP-binding protein	3.50
KIF53_RS13245	PAAR domain-containing protein	3.47
KIF53_RS13360	M15 family metallopeptidase	3.46
KIF53_RS07505	flagellar motor switch protein FliN	3.45
KIF53_RS03945	CRISPR-associated helicase/endonuclease Cas3	3.45
KIF53_RS04005	L-serine ammonia-lyase	3.45
KIF53_RS09805	hypothetical protein	3.44

KIF53_RS11695	chorismate-binding protein	3.43
KIF53_RS19355	amino acid adenylation domain-containing protein	3.43
KIF53_RS06790	ABC transporter permease subunit	3.42
KIF53_RS03950	type I-E CRISPR-associated protein Cse1/CasA	3.41
KIF53_RS13260	phage major tail tube protein	3.38
KIF53_RS19335	alpha/beta fold hydrolase	3.36
KIF53_RS00650	5-(carboxyamino)imidazole ribonucleotide mutase	3.34
KIF53_RS20220	chaperonin GroEL	3.34
KIF53_RS20765	indolepyruvate ferredoxin oxidoreductase family protein	3.32
KIF53_RS03960	type I-E CRISPR-associated protein Cas7/Cse4/CasC	3.32
KIF53_RS00655	5-(carboxyamino)imidazole ribonucleotide synthase	3.30
KIF53_RS15110	P-II family nitrogen regulator	3.30
KIF53_RS02575	elongation factor G	3.28
KIF53_RS18405	MarR family transcriptional regulator	3.28
KIF53_RS04885	aminomethyl-transferring glycine dehydrogenase	3.28
KIF53_RS07155	oxidoreductase	3.28
KIF53_RS01150	subunit	3.27
KIF53_RS16385	NCS2 family permease	3.23
KIF53_RS04965	acyl carrier protein	3.20
KIF53_RS17070	FAD-dependent oxidoreductase	3.20
KIF53_RS07315	integration host factor subunit beta	3.19
KIF53_RS10620	DUF3313 family protein	3.19
KIF53_RS02970	hypothetical protein	3.19
KIF53_RS14205	iron transporter	3.18
KIF53_RS01535	septal ring lytic transglycosylase RlpA family protein	3.16
KIF53_RS07500	flagellar type III secretion system pore protein FliP	3.15
KIF53_RS12190	F0F1 ATP synthase subunit beta	3.14
KIF53_RS00670	synthase	3.14
KIF53_RS13265	phage tail sheath subtilisin-like domain-containing protein	3.12
KIF53_RS21590	DUF6531 domain-containing protein	3.11
KIF53_RS21840	DUF805 domain-containing protein	3.10
KIF53_RS18410	energy transducer TonB	3.10
KIF53_RS01115	glycosyltransferase	3.09
KIF53_RS12180	F0F1 ATP synthase subunit alpha	3.09
KIF53_RS15065	co-chaperone GroES	3.08
KIF53_RS19340	cupin-like domain-containing protein	3.08
KIF53_RS02670	50S ribosomal protein L18	3.08
KIF53_RS15070	chaperonin GroEL	3.06
KIF53_RS21585	DUF2345 domain-containing protein	3.06
KIF53_RS13270	hypothetical protein	3.06
KIF53_RS02580	elongation factor Tu	3.03
KIF53_RS20225	co-chaperone GroES	3.00
KIF53_RS04740	phosphopyruvate hydratase	2.99
KIF53_RS12185	F0F1 ATP synthase subunit gamma	2.98
KIF53_RS17825	energy transducer TonB	2.97

KIF53_RS01140	hypothetical protein	2.96
KIF53_RS07310	30S ribosomal protein S1	2.96
KIF53_RS05660	hypothetical protein	2.95
KIF53_RS08985	acetyl-CoA carboxylase biotin carboxylase subunit	2.94
KIF53_RS13285	DUF2190 family protein	2.94
KIF53_RS02680	50S ribosomal protein L30	2.94
KIF53_RS02675	30S ribosomal protein S5	2.93
KIF53_RS16215	small subunit	2.93
KIF53_RS17940	DUF2059 domain-containing protein	2.93
KIF53_RS05180	fumarate reductase (quinol) flavoprotein subunit	2.93
KIF53_RS20390	methyltransferase domain-containing protein	2.91
KIF53_RS14110	GspH/FimT family pseudopilin	2.90
KIF53_RS02540	tRNA-Thr	2.90
KIF53_RS12560	BMP family ABC transporter substrate-binding protein	2.90
KIF53_RS03535	type 1 fimbrial protein	2.89
KIF53_RS02610	30S ribosomal protein S19	2.89
KIF53_RS02665	50S ribosomal protein L6	2.89
KIF53_RS01110	acyltransferase	2.88
KIF53_RS02830	acyl-CoA dehydrogenase family protein	2.87
KIF53_RS12175	F0F1 ATP synthase subunit delta	2.87
KIF53_RS17840	biopolymer transporter ExbD	2.87
KIF53_RS07495	flagellar biosynthesis protein FliQ	2.86
KIF53_RS05190	fumarate reductase	2.84
KIF53_RS17680	NERD domain-containing protein	2.84
KIF53_RS13280	DUF1320 domain-containing protein	2.82
KIF53_RS04880	glycine cleavage system protein GcvH	2.82
KIF53_RS15870	phosphoribosylformylglycinamidine synthase	2.81
KIF53_RS11700	queuosine precursor transporter	2.81
KIF53_RS04205	cytochrome c oxidase accessory protein CcoG	2.80
KIF53_RS08500	hypothetical protein	2.80
KIF53_RS05055	cystathionine beta-synthase	2.78
KIF53_RS01540	tRNA (uridine(34)/cytosine(34)/5-carboxymethylaminomethyluridine(34)-2'-O)-methyltransferase TrmL	2.78
KIF53_RS06905	flagellar hook-basal body complex protein	2.78
KIF53_RS02660	30S ribosomal protein S8	2.78
KIF53_RS05060	protein	2.77
KIF53_RS02545	50S ribosomal protein L10	2.74
KIF53_RS02270	orotate phosphoribosyltransferase	2.73
KIF53_RS22035	TerD family protein	2.71
KIF53_RS13250	hypothetical protein	2.70
KIF53_RS19325	3-hydroxyacyl-CoA dehydrogenase family protein	2.70
KIF53_RS13330	DUF3486 family protein	2.70
KIF53_RS02595	50S ribosomal protein L4	2.70
KIF53_RS11445	hypothetical protein	2.70

KIF53_RS00660	SEL1-like repeat protein	2.70
KIF53_RS21790	hypothetical protein	2.70
KIF53_RS12165	F0F1 ATP synthase subunit C	2.68
KIF53_RS02975	glycosyltransferase family 4 protein	2.67
KIF53_RS02605	50S ribosomal protein L2	2.67
KIF53_RS16955	fucose-binding lectin	2.66
KIF53_RS12170	F0F1 ATP synthase subunit B	2.66
KIF53_RS02655	30S ribosomal protein S14	2.65
KIF53_RS05525	carbohydrate porin	2.65
KIF53_RS09800	activating protein	2.64
KIF53_RS01980	hypothetical protein	2.64
KIF53_RS08190	nuclear transport factor 2 family protein	2.63
KIF53_RS04970	beta-ketoacyl-ACP synthase II	2.63
KIF53_RS14595	rhodoquinone biosynthesis methyltransferase RquA	2.62
KIF53_RS01155	peptidase domain-containing ABC transporter	2.61
KIF53_RS07480	flagellar biosynthesis protein FlhA	2.61
KIF53_RS06910	flagellar basal body rod protein FlgF	2.60
KIF53_RS08490	ADP-forming succinate--CoA ligase subunit beta	2.59
KIF53_RS05030	(2Fe-2S)-binding protein	2.59
KIF53_RS06960	cytochrome c	2.59
KIF53_RS06000	tRNA-Asp	2.58
KIF53_RS00960	proton-translocating transhydrogenase family protein	2.58
KIF53_RS11805	DUF2860 family protein	2.58
KIF53_RS04950	ketoacyl-ACP synthase III	2.57
KIF53_RS02615	50S ribosomal protein L22	2.56
KIF53_RS09795	anaerobic ribonucleoside-triphosphate reductase	2.55
KIF53_RS06895	flagellar basal body rod protein FlgC	2.55
KIF53_RS11255	glutamate--ammonia ligase	2.55
KIF53_RS06310	GNAT family N-acetyltransferase	2.54
KIF53_RS03975	type I-E CRISPR-associated endonuclease Cas1e	2.53
KIF53_RS21355	Smr/MutS family protein	2.53
KIF53_RS03060	outer membrane beta-barrel protein	2.53
KIF53_RS21595	hypothetical protein	2.53
KIF53_RS02880	class I SAM-dependent methyltransferase	2.52
KIF53_RS17440	UDP-2,3-diacylglucosamine diphosphatase	2.50
KIF53_RS02035	acid phosphatase	2.47
KIF53_RS02550	50S ribosomal protein L7/L12	2.46
KIF53_RS10935	GbsR/MarR family transcriptional regulator	2.46
KIF53_RS06900	flagellar basal body rod modification protein FlgD	2.46
KIF53_RS06055	cobyrinate a,c-diamide synthase	2.46
KIF53_RS16910	GyrI-like domain-containing protein	2.45
KIF53_RS08485	succinate--CoA ligase subunit alpha	2.45
KIF53_RS04195	LysR family transcriptional regulator	2.44
KIF53_RS10945	cytochrome d ubiquinol oxidase subunit II	2.44
KIF53_RS21290	adenylosuccinate synthase	2.44

KIF53_RS13745	acetyl-CoA C-acyltransferase	2.43
KIF53_RS02885	sulfotransferase	2.43
KIF53_RS12915	BMP family ABC transporter substrate-binding protein	2.42
KIF53_RS04010	HAAAP family serine/threonine permease	2.42
KIF53_RS03980	type I-E CRISPR-associated endoribonuclease Cas2	2.41
KIF53_RS06660	protoporphyrinogen oxidase	2.41
KIF53_RS13740	MerR family DNA-binding transcriptional regulator	2.41
KIF53_RS00665	DNA alkylation repair protein	2.40
KIF53_RS19725	enterobactin transporter EntS	2.40
KIF53_RS15105	ammonium transporter	2.39
KIF53_RS19960	nicotinate-nucleotide adenylyltransferase	2.39
KIF53_RS18715	tRNA-Pro	2.39
KIF53_RS08975	type II 3-dehydroquinate dehydratase	2.38
KIF53_RS05195	fumarate reductase subunit FrdD	2.38
KIF53_RS02535	50S ribosomal protein L1	2.36
KIF53_RS05475	2OG-Fe dioxygenase family protein	2.36
KIF53_RS11710	hypothetical protein	2.36
KIF53_RS02600	50S ribosomal protein L23	2.36
KIF53_RS10510	QueF	2.36
KIF53_RS04285	polyribonucleotide nucleotidyltransferase	2.35
KIF53_RS12815	hypothetical protein	2.34
KIF53_RS16905	tetratricopeptide repeat protein	2.33
KIF53_RS02935	DUF4337 domain-containing protein	2.33
KIF53_RS07055	hypothetical protein	2.33
KIF53_RS02530	50S ribosomal protein L11	2.33
KIF53_RS09770	NAD(P)H-quinone oxidoreductase	2.32
KIF53_RS04875	glycine cleavage system aminomethyltransferase GcvT	2.32
KIF53_RS15835	DUF697 domain-containing protein	2.32
KIF53_RS04340	iron ABC transporter permease	2.31
KIF53_RS02690	preprotein translocase subunit SecY	2.30
KIF53_RS03905	EAL domain-containing protein	2.29
KIF53_RS06925	flagellar basal body P-ring protein FlgI	2.29
KIF53_RS10940	cytochrome ubiquinol oxidase subunit I	2.27
KIF53_RS02715	30S ribosomal protein S4	2.27
KIF53_RS07530	flagellar assembly protein H	2.26
KIF53_RS17425	OmpP1/FadL family transporter	2.26
KIF53_RS11770	DUF3619 family protein	2.25
KIF53_RS09615	hypothetical protein	2.25
KIF53_RS02950	oligosaccharide flippase family protein	2.25
KIF53_RS07535	flagellar protein export ATPase FliI	2.25
KIF53_RS11060	anaerobic C4-dicarboxylate transporter	2.24
KIF53_RS02620	30S ribosomal protein S3	2.24
KIF53_RS07515	flagellar hook-basal body complex protein FliE	2.24
KIF53_RS12195	F0F1 ATP synthase subunit epsilon	2.24
KIF53_RS04945	phosphate acyltransferase PlsX	2.23

KIF53_RS17835	MotA/TolQ/ExbB proton channel family protein	2.23
KIF53_RS13735	3-hydroxybutyryl-CoA dehydrogenase	2.22
KIF53_RS02960	glycosyltransferase family 4 protein	2.22
KIF53_RS17140	hypothetical protein	2.22
KIF53_RS08495	dihydrolipoyl dehydrogenase	2.22
KIF53_RS19555	phosphonate C-P lyase system protein PhnG	2.22
KIF53_RS02310	chitinase	2.21
KIF53_RS02870	sulfotransferase family 2 domain-containing protein	2.21
KIF53_RS04350	DUF2218 domain-containing protein	2.21
KIF53_RS06915	flagellar basal-body rod protein FlgG	2.20
KIF53_RS05610	hypothetical protein	2.20
KIF53_RS02570	30S ribosomal protein S7	2.20
KIF53_RS07120	NAD-glutamate dehydrogenase	2.19
KIF53_RS09855	phosphatidate cytidylyltransferase	2.19
KIF53_RS01850	GNAT family N-acetyltransferase	2.19
KIF53_RS01635	copper chaperone PCu(A)C	2.19
KIF53_RS10515	CPBP family intramembrane metalloprotease	2.19
KIF53_RS06890	flagellar basal body rod protein FlgB	2.18
KIF53_RS16005	hypothetical protein	2.17
KIF53_RS22550	type VI secretion system tip protein VgrG	2.17
KIF53_RS02500	tRNA-Gly	2.17
KIF53_RS02945	DegT/DnrJ/EryC1/StrS family aminotransferase	2.17
KIF53_RS15775	metal-dependent hydrolase	2.16
KIF53_RS05745	sugar MFS transporter	2.15
KIF53_RS15825	Ldh family oxidoreductase	2.15
KIF53_RS09860	1-deoxy-D-xylulose-5-phosphate reductoisomerase	2.15
KIF53_RS02720	DNA-directed RNA polymerase subunit alpha	2.14
KIF53_RS09905	DNA-deoxyinosine glycosylase	2.14
KIF53_RS22040	VWA domain-containing protein	2.14
KIF53_RS06060	cob(I)yrinic acid a,c-diamide adenosyltransferase	2.13
KIF53_RS07555	hypothetical protein	2.13
KIF53_RS00575	isopenicillin N synthase family oxygenase	2.13
KIF53_RS07440	ribose ABC transporter substrate-binding protein RbsB	2.13
KIF53_RS11335	DUF934 domain-containing protein	2.12
KIF53_RS13275	Gp37 family protein	2.12
KIF53_RS02965	glycosyltransferase family 4 protein	2.12
KIF53_RS06085	precorrin-3B C(17)-methyltransferase	2.11
KIF53_RS06095	precorrin-4 C(11)-methyltransferase	2.11
KIF53_RS00965	beta	2.11
KIF53_RS13645	metal-dependent hydrolase	2.11
KIF53_RS12160	F0F1 ATP synthase subunit A	2.10
KIF53_RS01355	hypothetical protein	2.10
KIF53_RS07540	flagellar export protein FliJ	2.10
KIF53_RS02890	tRNA-Met	2.10
KIF53_RS12920	MATE family efflux transporter	2.10

KIF53_RS15815	hypothetical protein	2.09
KIF53_RS11435	dihydroorotate oxidase	2.09
KIF53_RS12570	ABC transporter permease	2.09
KIF53_RS20600	acyl-CoA thioesterase	2.09
KIF53_RS22580	type VI secretion system Vgr family protein	2.09
KIF53_RS22005	hypothetical protein	2.09
KIF53_RS12575	ABC transporter permease	2.08
KIF53_RS13730	dehydrogenase	2.08
KIF53_RS04335	ABC transporter ATP-binding protein	2.08
KIF53_RS00820	transketolase	2.08
KIF53_RS10395	peptidoglycan DD-metalloendopeptidase family protein	2.07
KIF53_RS02505	tRNA-Thr	2.07
KIF53_RS07685	sigma-70 family RNA polymerase sigma factor	2.07
KIF53_RS06065	iron ABC transporter permease	2.06
KIF53_RS05240	DUF456 family protein	2.06
KIF53_RS04960	3-oxoacyl-ACP reductase FabG	2.06
KIF53_RS07485	flagellar type III secretion system protein FlhB	2.05
KIF53_RS22030	hypothetical protein	2.05
KIF53_RS16885	nuclear transport factor 2 family protein	2.05
KIF53_RS16330	arginine-ornithine antiporter	2.05
KIF53_RS20805	glutathione transferase GstA	2.05
KIF53_RS03955	type I-E CRISPR-associated protein Cse2/CasB	2.04
KIF53_RS11450	catalase	2.04
KIF53_RS03920	elongation factor P	2.04
KIF53_RS16605	phosphatase PAP2 family protein	2.04
KIF53_RS16020	formate dehydrogenase accessory protein FdhE	2.04
KIF53_RS07520	flagellar M-ring protein FliF	2.03
KIF53_RS19230	MFS transporter	2.03
KIF53_RS13650	holo-ACP synthase	2.03
KIF53_RS02650	50S ribosomal protein L5	2.03
KIF53_RS09760	cbb3-type cytochrome oxidase assembly protein CcoS	2.03
KIF53_RS04355	TonB-dependent receptor	2.03
KIF53_RS06580	nucleotide exchange factor GrpE	2.03
KIF53_RS21785	hypothetical protein	2.02
KIF53_RS05680	aminotransferase class III-fold pyridoxal phosphate-dependent enzyme	2.02
KIF53_RS06080	GTP-binding protein	2.02
KIF53_RS07525	flagellar motor switch protein FliG	2.02
KIF53_RS08250	methyl-accepting chemotaxis protein	2.02
KIF53_RS16130	MaoC family dehydratase	2.01
KIF53_RS03760	preprotein translocase subunit YajC	2.01
KIF53_RS01520	cytochrome c biogenesis protein ResB	2.01
KIF53_RS02980	DegT/DnrJ/EryC1/StrS family aminotransferase	2.01
KIF53_RS02925	YdcF family protein	2.00

C. subtsugae genes activated by C8-HSL

Locus tag corresponding with accession NZ_JAHDTB000000000.1	Predicted gene function	Log_2 fold change compared with no AHLs
KIF53_RS16340	cyanide-forming glycine dehydrogenase subunit HcnA	6.41
KIF53_RS20400	cyanide-forming glycine dehydrogenase subunit HcnC	5.61
KIF53_RS02310	ornithine carbamoyltransferase	5.22
KIF53_RS06310	hybrid non-ribosomal peptide synthetase/type I polyketide synthase	4.97
KIF53_RS15105	chitinase	4.71
KIF53_RS06795	GNAT family N-acetyltransferase	3.86
KIF53_RS20405	ammonium transporter	3.97
KIF53_RS21025	ABC transporter substrate-binding protein	4.58
KIF53_RS06805	amino acid adenylation domain-containing protein	4.80
KIF53_RS05635	alpha/beta fold hydrolase	4.65
KIF53_RS15685	NAD(P)/FAD-dependent oxidoreductase	6.95
KIF53_RS05525	FAD-dependent oxidoreductase	4.14
KIF53_RS16345	M9 family metallopeptidase N-terminal domain-containing protein	3.67
KIF53_RS04205	carbohydrate porin	3.16
KIF53_RS14555	carbamate kinase	3.93
KIF53_RS13810	cytochrome c oxidase accessory protein CcoG	3.35
KIF53_RS16335	hybrid non-ribosomal peptide synthetase/type I polyketide synthase	3.29
KIF53_RS06780	activator protein	4.40
KIF53_RS02825	arginine deiminase	3.95
KIF53_RS20410	ABC transporter ATP-binding protein	4.43
KIF53_RS21040	hydrogenase maturation protein	3.18
KIF53_RS21035	non-ribosomal peptide synthetase	4.04
KIF53_RS15785	hypothetical protein	4.75
KIF53_RS15110	polysaccharide pyruvyl transferase family protein	3.91
KIF53_RS05190	methyl-accepting chemotaxis protein	2.60
KIF53_RS05180	P-II family nitrogen regulator	3.83
KIF53_RS11805	fumarate reductase	3.17
KIF53_RS17975	fumarate reductase (quinol) flavoprotein subunit	2.70
KIF53_RS14595	DUF2860 family protein	2.53
KIF53_RS01130	aspartate carbamoyltransferase	3.03
KIF53_RS03060	rhodoquinone biosynthesis methyltransferase RquA	2.70
KIF53_RS16905	transketolase	3.08
KIF53_RS21030	outer membrane beta-barrel protein	3.38
KIF53_RS10620	tetratricopeptide repeat protein	2.60

KIF53_RS18600	4'-phosphopantetheinyl transferase superfamily protein	3.58
KIF53_RS10945	DUF3313 family protein	2.82
KIF53_RS01150	LysE family translocator	2.91
KIF53_RS13710	cytochrome d ubiquinol oxidase subunit II	3.05
KIF53_RS17525	HlyD family efflux transporter periplasmic adaptor subunit	2.95
KIF53_RS13945	3-hydroxyisobutyrate dehydrogenase	2.29
KIF53_RS13960	ssDNA-binding domain-containing protein	3.06
KIF53_RS13880	hypothetical protein	2.65
KIF53_RS16225	Flp pilus assembly complex ATPase component TadA	2.41
KIF53_RS13925	hypothetical protein	2.61
KIF53_RS05195	carbamoyl-phosphate synthase large subunit	2.71
KIF53_RS17485	PilN family type IVB pilus formation outer membrane protein	2.66
KIF53_RS17680	fumarate reductase subunit FrdD	2.88
KIF53_RS09615	DotA/TraY family protein	2.45
KIF53_RS20765	NERD domain-containing protein	2.66
KIF53_RS11255	hypothetical protein	3.10
KIF53_RS20415	indolepyruvate ferredoxin oxidoreductase family protein	2.52
KIF53_RS20465	glutamate--ammonia ligase	3.21
KIF53_RS10940	acyltransferase domain-containing protein	3.73
KIF53_RS01145	GNAT family N-acetyltransferase	2.46
KIF53_RS17060	cytochrome ubiquinol oxidase subunit I	2.82
KIF53_RS16910	hypothetical protein	3.93
KIF53_RS17075	magnesium-translocating P-type ATPase	2.47
KIF53_RS05185	GyrI-like domain-containing protein	2.86
KIF53_RS11400	porin	3.25
KIF53_RS13855	succinate dehydrogenase/fumarate reductase iron-sulfur subunit	4.07
KIF53_RS00720	histone deacetylase family protein	2.05
KIF53_RS21000	hypothetical protein	2.44
KIF53_RS01125	depolymerase	2.77
KIF53_RS18595	hypothetical protein	2.96
KIF53_RS14025	hypothetical protein	3.15
KIF53_RS21015	PLP-dependent aminotransferase family protein	2.36
KIF53_RS15070	YeiH family putative sulfate export transporter	2.21
KIF53_RS07545	hypothetical protein	3.13
KIF53_RS12920	chaperonin GroEL	2.84
KIF53_RS13860	flagellar filament capping protein FliD	2.22
KIF53_RS20390	MATE family efflux transporter	2.30
KIF53_RS17750	hypothetical protein	2.47
KIF53_RS21840	methyltransferase domain-containing protein	2.79
KIF53_RS04885	L-threonine dehydrogenase	2.50
KIF53_RS17520	DUF805 domain-containing protein	2.61
KIF53_RS05640	aminomethyl-transferring glycine dehydrogenase	2.40

KIF53_RS06790	hypothetical protein	3.22
KIF53_RS13715	iminophenyl-pyruvate dimer synthase VioB	2.51
KIF53_RS15405	ABC transporter permease subunit	2.81
KIF53_RS16915	enoyl-CoA hydratase/isomerase family protein	2.29
KIF53_RS19365	hypothetical protein	2.50
KIF53_RS16820	hydroxymethylglutaryl-CoA lyase	2.24
KIF53_RS13920	type I polyketide synthase	2.56
KIF53_RS21010	M4 family metallopeptidase	2.76
KIF53_RS01140	toxin co-regulated pilus biosynthesis Q family protein	2.87
KIF53_RS01120	SDR family NAD(P)-dependent oxidoreductase	4.10
KIF53_RS00965	hypothetical protein	2.56
KIF53_RS04020	glycosyltransferase family 2 protein	2.89
KIF53_RS17515	NAD(P)(+) transhydrogenase (Re/Si-specific) subunit beta	2.03
KIF53_RS07155	formate C-acetyltransferase	2.20
KIF53_RS01860	polymer-forming cytoskeletal protein	2.34
KIF53_RS06785	oxidoreductase	2.73
KIF53_RS09620	long-chain-fatty-acyl-CoA reductase	2.10
KIF53_RS16955	ABC transporter permease subunit	3.49
KIF53_RS16900	beta-ketoacyl-ACP synthase	2.24
KIF53_RS19375	fucose-binding lectin	2.10
KIF53_RS13940	acetyl/propionyl/methylcrotonyl-CoA carboxylase subunit alpha	2.07
KIF53_RS00955	ACP S-malonyltransferase	2.69
KIF53_RS01960	Flp pilus assembly complex ATPase component TadA	2.51
KIF53_RS17530	Re/Si-specific NAD(P)(+) transhydrogenase subunit alpha	2.08
KIF53_RS15570	universal stress protein	2.36
KIF53_RS01110	hypothetical protein	2.44
KIF53_RS03105	hypothetical protein	2.35
KIF53_RS19370	acyltransferase	2.40
KIF53_RS12190	cbb3-type cytochrome c oxidase subunit 3	2.12
KIF53_RS13895	AMP-binding protein	2.55
KIF53_RS13900	F0F1 ATP synthase subunit beta	2.39
KIF53_RS13930	type IV secretory system conjugative DNA transfer family protein	2.42
KIF53_RS16325	Flp pilus assembly complex ATPase component TadA	2.47
KIF53_RS14685	type 4b pilus protein PilO2	2.61
KIF53_RS13955	DUF3149 domain-containing protein	3.74
KIF53_RS09025	U32 family peptidase	2.25
KIF53_RS13815	hypothetical protein	2.09
KIF53_RS08485	PTS glucose transporter subunit IIBC	2.07
KIF53_RS09020	helix-turn-helix domain-containing protein	2.01
KIF53_RS05540	succinate--CoA ligase subunit alpha	2.13
KIF53_RS02580	phosphoenolpyruvate--protein phosphotransferase	2.06

KIF53_RS00960	hypothetical protein	2.10
KIF53_RS01115	elongation factor Tu	2.23
KIF53_RS21020	proton-translocating transhydrogenase family protein	2.39
KIF53_RS13865	glycosyltransferase	2.04
KIF53_RS15065	PfaD family polyunsaturated fatty acid/polyketide biosynthesis protein	2.97
KIF53_RS13730	hypothetical protein	3.05
KIF53_RS07290	co-chaperone GroES	2.24
KIF53_RS22565	CoA-acylating methylmalonate-semialdehyde dehydrogenase	2.07
KIF53_RS15830	cytochrome b	2.10
KIF53_RS22325	hypothetical protein	2.28
KIF53_RS17610	hypothetical protein	2.34
KIF53_RS06960	hypothetical protein	2.39
KIF53_RS05655	phenylacetate-CoA oxygenase subunit PaaI	2.15
KIF53_RS09625	cytochrome c	2.05
KIF53_RS03110	violacein biosynthesis enzyme VioE	2.33
KIF53_RS13875	3-oxoacyl-ACP reductase FabG	2.80
KIF53_RS19360	cytochrome-c oxidase, cbb3-type subunit II	2.03
KIF53_RS21790	hypothetical protein	2.42
KIF53_RS13850	serine hydrolase	2.09
KIF53_RS22560	hypothetical protein	2.12
KIF53_RS09630	DotI/IcmL family type IV secretion protein	2.22
KIF53_RS19555	hypothetical protein	2.08
KIF53_RS14680	hotdog family protein	2.43
KIF53_RS12980	phosphonate C-P lyase system protein PhnG	2.01
KIF53_RS05645	U32 family peptidase	2.32
KIF53_RS02970	type III secretion system inner rod subunit SctI	2.01
KIF53_RS14110	FAD-dependent monooxygenase	2.22
	hypothetical protein	2.07
	GspH/FimT family pseudopilin	2.02
